# Gateway-compatible vectors for functional analysis of proteins in cell type specific manner

**DOI:** 10.1186/s13007-020-00635-z

**Published:** 2020-07-06

**Authors:** Liu Zhang, Yang Zhao, Haiyan Liang, Xugang Li, Kimberly L. Gallagher, Shuang Wu

**Affiliations:** 1 College of Life Sciences, FAFU-UCR Joint Center and Fujian Provincial Key Laboratory of Haixia Applied Plant Systems Biology, Fujian Agriculture and Forestry University, Fuzhou, China; 2 College of Resource and Environment, Fujian Agriculture and Forestry University, Fuzhou, China; 3 State Key Laboratory of Crop Biology, College of Life Sciences, Shandong Agricultural University, Tai’an, China; 4 University of Pennsylvania, Philadelphia, PA 19104 USA

**Keywords:** Fluorescent proteins, Photoconversion, Gateway vectors, Cell-specific expression, Arabidopsis

## Abstract

**Background:**

Genetically encoded fluorescent proteins are often used to label proteins and study protein function and localization in vivo. Traditional cloning methods mediated by restriction digestion and ligation are time-consuming and sometimes difficult due to the lack of suitable restriction sites. Invitrogen developed the Gateway cloning system based on the site-specific DNA recombination, which allows for digestion-free cloning. Most gateway destination vectors available for use in plants employ either the 35S or ubiquitin promoters, which confer high-level, ubiquitous expression. There are far fewer options for moderate, cell-type specific expression.

**Results:**

Here we report on the construction of a Gateway-compatible cloning system (SWU vectors) to rapidly tag various proteins and express them in a cell-type specific manner in plants. We tested the SWU vectors using the HISTONE (H2B) coding sequence in stable transgenic plants.

**Conclusions:**

The SWU vectors are a valuable tool for low cost, high efficiency functional analysis of proteins of interest in specific cell types in the *Arabidopsis* root.

## Background

Since the invention of microscopy, imaging has been one of the central focuses of biology. The production and use of fluorescently labelled antibodies enabled the visualization of specific cellular components and proteins in situ. However, the examination of fixed and stained cells provides only a static snapshot of protein localization. The discovery of genetically encoded fluorescent proteins (FPs) has revolutionized cell biology by allowing for the direct fusion of a fluorescent protein to a protein of interest, thus enabling the non-destructive localization and tracking of the fluorescently labeled protein in vivo. A broad range of FPs have been produced, spanning nearly the entire visible spectrum [1]. The combination of different fluorescent markers enables simultaneous detection of multiple proteins within a cell; striking examples of this technique are presented by Livet et al. (2007 and at http://cbs.fas.harvard.edu/science/connectome-project/brainbow). In addition to cyan, green, yellow and red fluorescing proteins exemplified by CFP (or cearulean), GFP, YFP and RFP (or mCherry) respectively, several photoconvertible proteins (proteins can be transformed from one color to another in response to a specific wavelength of light) are also available [2–6]. For example, Dendra2 isolated from coral is green in its native state and switches to red when irradiated by 405 nm laser. Both Dendra2 and mEosFP have proved to be powerful tools in the study protein dynamics, protein trafficking and protein–protein interaction [2–6].

The utilization of genetically encoded fluorescent proteins (FPs) relies on DNA cloning techniques that enable the in-frame fusion of the coding sequence of the FP to the coding sequence of the protein of interest within a DNA cassette that contains a promoter appropriate for expression in the system of interest. Many studies in plant systems utilize constitutive promoters (e.g. 35S and ubiquitin promoters). This is particularly useful when the native promoter has not been determined or when over expression is the goal. In addition, some techniques such as BIFC, which are performed in heterologous plant systems are benefitted by the broad host range that the 35S or ubiquitin promoters provide [6–9]. Despite their wide usage, constitutive promoters have their drawbacks. Proteins that are overexpressed may promiscuously associate with cellular compartments or mislocalize. In addition, the phenotypes generated via overexpression may confound imaging. Two of the biggest problems that we have encountered with the use of constitutive promoters in transgenic lines are the issues of transgene silencing and co-suppression (Additional file 1: Figure S1). For the 35S and the UBQ10 promoters, we have found this to be particularly true in the root meristem. For these reasons, we often choose to use promoters that show cell or tissue specific patterns of gene whenever possible. Even when the regulatory sequences of a gene are not known, it is often possible to query its pattern of expression in publically accessible databases (e.g. AtGENExpress). Known cell specific promoters therefore could be used to approximate normal gene expression, which may be preferable to constitutive expression. To this end, we have developed a flexible series of Gateway enabled plant expression vectors for cell specific gene transcription in the root of *Arabidopsis thaliana.*

The destination vectors (described in detail below) use a modified pGreenBarT backbone in which the attR4/attR3 cassette is replaced with an attR1/attR2 cassette to alleviate the need for 3-way reactions and facilitate high-throughput cloning. All destination vectors contain a cell-type specific promoter and a florescent tag for in-frame fusion to the C-terminus of the gene of interest. These destination vectors can be used for a wide range of purposes including protein localization, protein function analysis, protein dynamics, protein movement and protein/protein interactions. As we include a multi-cloning site at the 5′ end of gateway cassette, the vectors can also be easily modified by adding other promoters that are active in leaves, flowers and other tissues.

## Results

### Constructing multi-color gateway-compatible binary vectors for cell-type specific expression

**Fig. 1:**
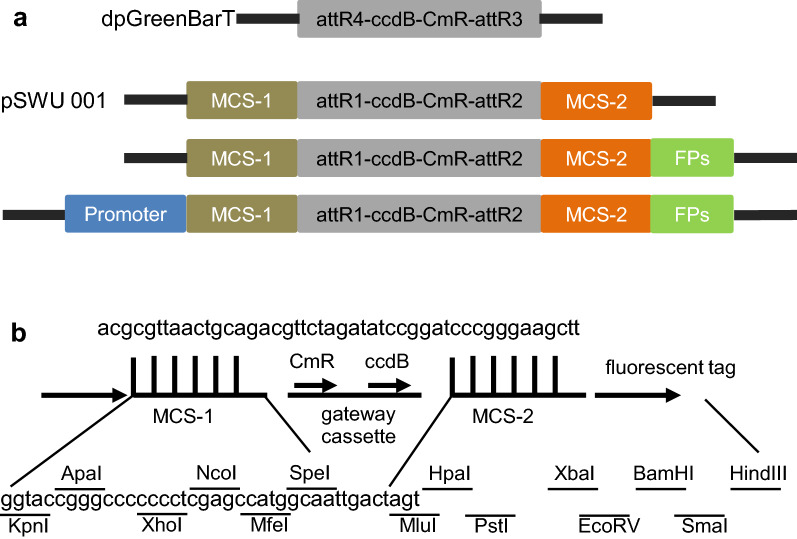
Cloning procedure of the intermediate vectors. **a** The backbone of dpGreenBarT was modified with attR4/attR3 gateway cassette replaced by attR1/attR2 gateway cassette being flanked by two multi-cloning sites (MCS-1 and MCS-2). The resulting vector was named as pSWU001. Different fluorescent proteins (FPs) and cell-specific promoters (labelled as promoter) were sequentially introduced into MCS-2 and MCS-1 of pSWU001 respectively. The complete sequences for MCS-1 and MCS-2 are shown in **b**

**Fig. 2:**
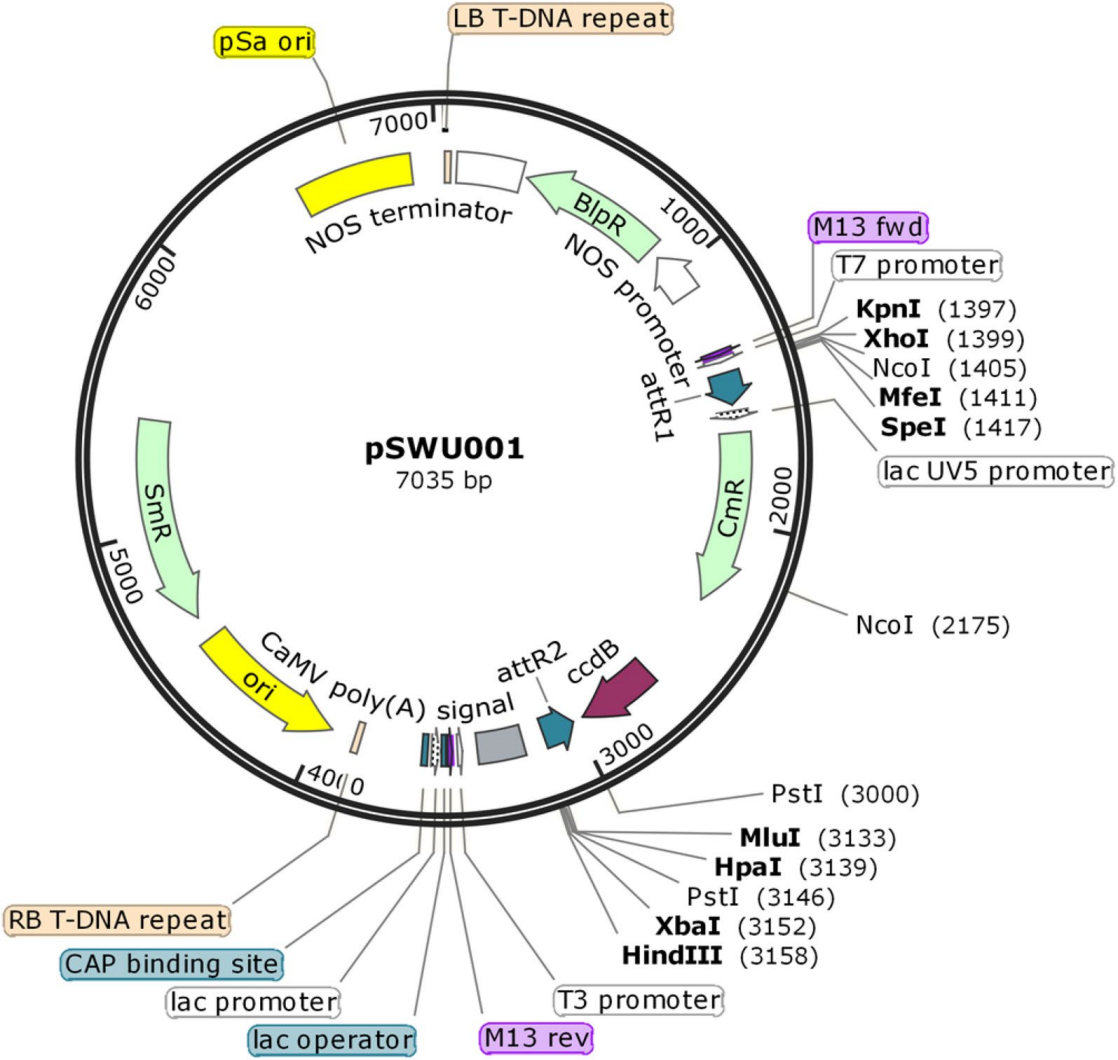
Map of the intermediate vector pSWU001. Restriction enzyme cutting sites are indicated. Features including antibiotic resistant genes, terminators and gateway cassette are shown

We chose to use the pGreenBarT binary vector as the backbone for our constructs because of its small size (7035 bps), and because it confers spectinomycin resistance, which is different from the commonly used kanamycin resistance in most pDONR entry clones. As previously described, the pGreenBarT destination vector was designed for multisite gateway cloning [10]. As such, pGreenBarT contains the ccdB and the CmR genes flanked by attR4 and attR3 recombination sites that react simultaneously with three different entry clones—generally pDONR P4-P1R containing the promoter fragment, pDONR 221 containing the gene of interest and pDONR P2R-P3 encodes a protein tag—to create a construct that drives marked, promoter specific expression of the gene of interest. In our hands, however the efficiency of the three-way recombination reaction is often low. In addition, these reactions require LR clonase II plus and commercial competent cells such as TOP10 cells (Add supplier), which are a significant added expense and limit the production of recombinant fusion proteins on a large scale. To avoid those hurdles, we adapted this vector by replacing attR4/attR3 gateway cassette with attR1/attR2 sites flanked by two multiple cloning sites (MCS) (Fig. 1a). To do this, the attR1/attR2 gateway cassette was amplified from a previously reported vector, pMDC7, with the first set of primers including the half of the two MCS at each end [11]. The product was then reamplified using a second set of primers, which included the MCS and introduced into pGreenBarT (see “Method” section for details). The obtained intermediate vector (pSWU001) and the sequence for both MCS are shown in Fig. 1. The whole plasmid map is shown in Fig. 2a.

**Fig. 3:**
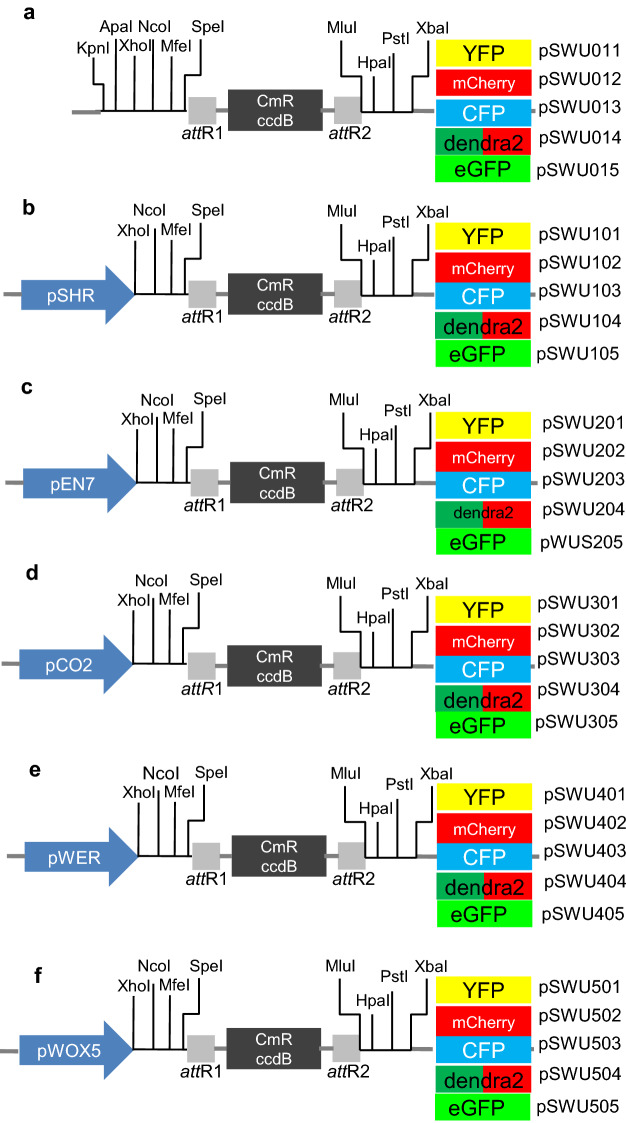
A summary of available Gateway-compatible vectors. **a** Modified pSWU001 vectors with fluorescent proteins (FPs) inserted into the MCS-2 at 3 terminus of the gateway cassette. **b** The vectors shown in **a** were further modified by adding pSHR into MCS-1 at 5 terminus of the gateway cassette. **c** The vectors shown in **a** were further modified by adding pEN7 into MCS-1 at 5 terminus of the gateway cassette. **d** The vectors shown in **a** were further modified by adding pCO2 into MCS-1 at 5 terminus of the gateway cassette. **e** The vectors shown in **a** were further modified by adding pWER into MCS-1 at 5 terminus of the gateway cassette. **f** The vectors shown in **a** were further modified by adding pWOX5 into MCS-1 at 5 terminus of the gateway cassette

To generate the specific expression in stele, endodermis, cortex, epidermis and QC cells, we cloned the promoters of *SHORT*-*ROOT* (*SHR*), *ENDODERMIS7* (*EN7*), *CORTEX2* (*CO2*), *WEREWOLF* (*WER*) and *WUSCHEL*-*LIKE HOMEOBOX 5* (*WOX5*) into the MCS upstream of gateway cassette (Fig. 3).

As the expression of *pSHR* was excluded from the phloem, we also constructed the promoter of *Cytokinin Response1* (*pWOL*) that has a strong expression pattern in all vascular tissues in root meristem (Additional file 2: Figure S2a and b) Cytokinin Response1. Down-stream of the attR1/attR2 each vector contains either Yellow Fluorescent Protein (YFP), monomeric Cherry (mCherry), Cyan Fluorescent Protein (CFP), Dendra2 or ER localized Green Fluorescent Protein (erGFP). These fluorescent proteins cover a wide spectrum of visible light; additionally, Dendra2 is photoconvertible.

### Imaging fusion proteins in stable transformed plants

**Fig. 4:**
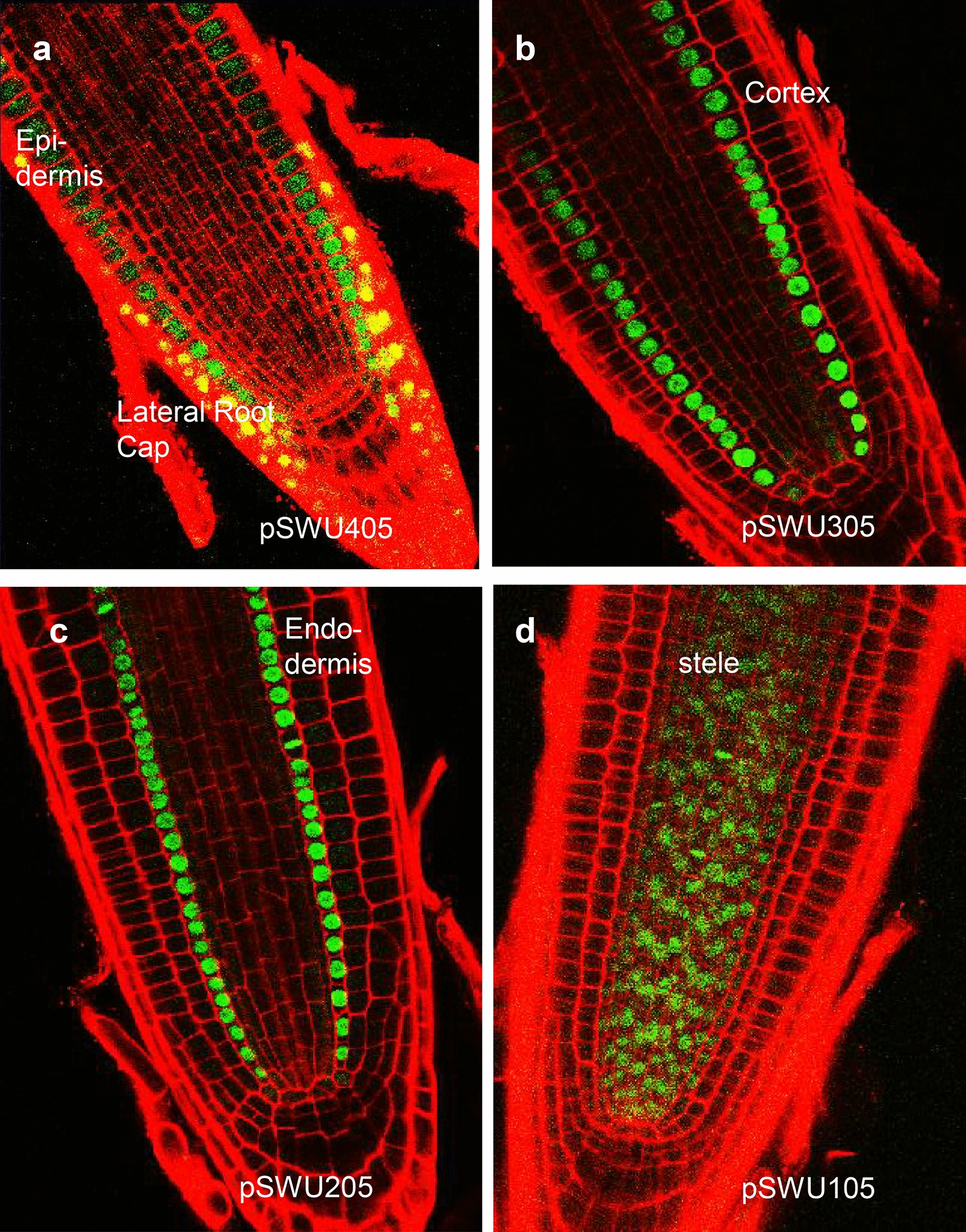
Expression of H2B-YFP in different cell layers of Arabidopsis roots. The primary root meristems were counterstained using PI (red) to show the cellular structure. Confocal images show that H2B-YFP exhibits nuclear localization in a cell specific manner. **a**–**d** The confocal images are respectively of *pWER:H2B*-*YFP* (**a**), *pCO2:H2B*-*YFP* (**b**), *pEN7:H2B*-*YFP* (**c**), *pSHR:H2B*-*YFP* (**d**)

**Fig. 5:**
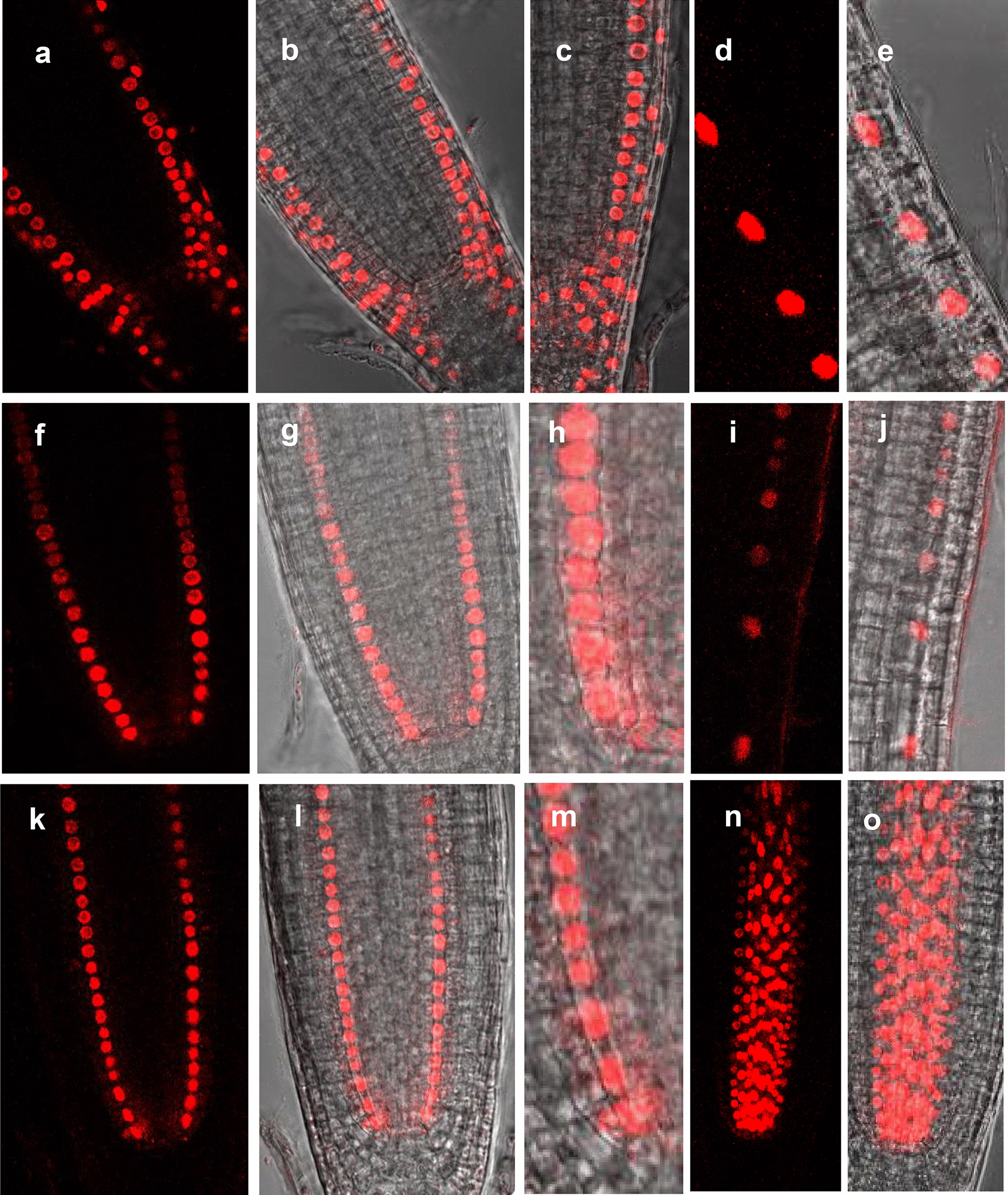
Expression of H2B-mCherry in different cell layers of Arabidopsis roots. **a**–**e***pWER:H2B*-*mCherry* exhibits epidermal and lateral root cap expression in meristem (**a**–**c**); and only epidermal expression in expansion zone (**d** and **e**). **b**, **c** and **e** are the overlay of red channel and DIC. **f**–**j***pCO2:H2B*-*mCherry* exhibits cortex expression in both meristem (**f**–**h**); and in expansion zone (**i** and **j**). **g**, **h** and **j** are the overlay of red channel and DIC. **k**–**m***pEN7:H2B*-*mCherry* exhibits expression in endodermis. **l**, **m** are the overlay of red channel and DIC. (n and o) *pSHR:H2B*-*mCherry* exhibits expression in stele. **o** Is the overlay of red channel and DIC

As proof of concept, we tested the functionality of the pSWU vectors in-planta using the histone, H2B (At3g45980) coding sequence [4]. The H2B protein localizes to the nucleus and does not move between cells, making the protein a widely used cell-autonomous marker of gene expression [8, 12]. Here using our expression system, we expanded the markers into different colors, which is particularly useful when imaging more than one protein at a time. As shown in Fig. 4, H2B-YFP was localized to the root cell nuclei; the expression pattern of H2B-YFP in each of these lines was consistent with the published patterns of expression for these promoters in the *Arabidopsis* root. The cellular organization of the root meristem was visualized using propidium iodide (PI) staining (Fig. 4). Because of overlap in the emission spectra of mCherry and PI, the root cellular patterning in the H2B-mCherry lines is shown using differential interference contrast (DIC) microscopy Fig. 5. In both the H2B-mCherry and H2B-YFP lines the localization of H2B and expression was consistent with published results. Furthermore, we saw no evidence for transgene silencing or partial suppression of expression (e.g. silencing in the initial cells) that would affect the domain of activity.

**Fig. 6:**
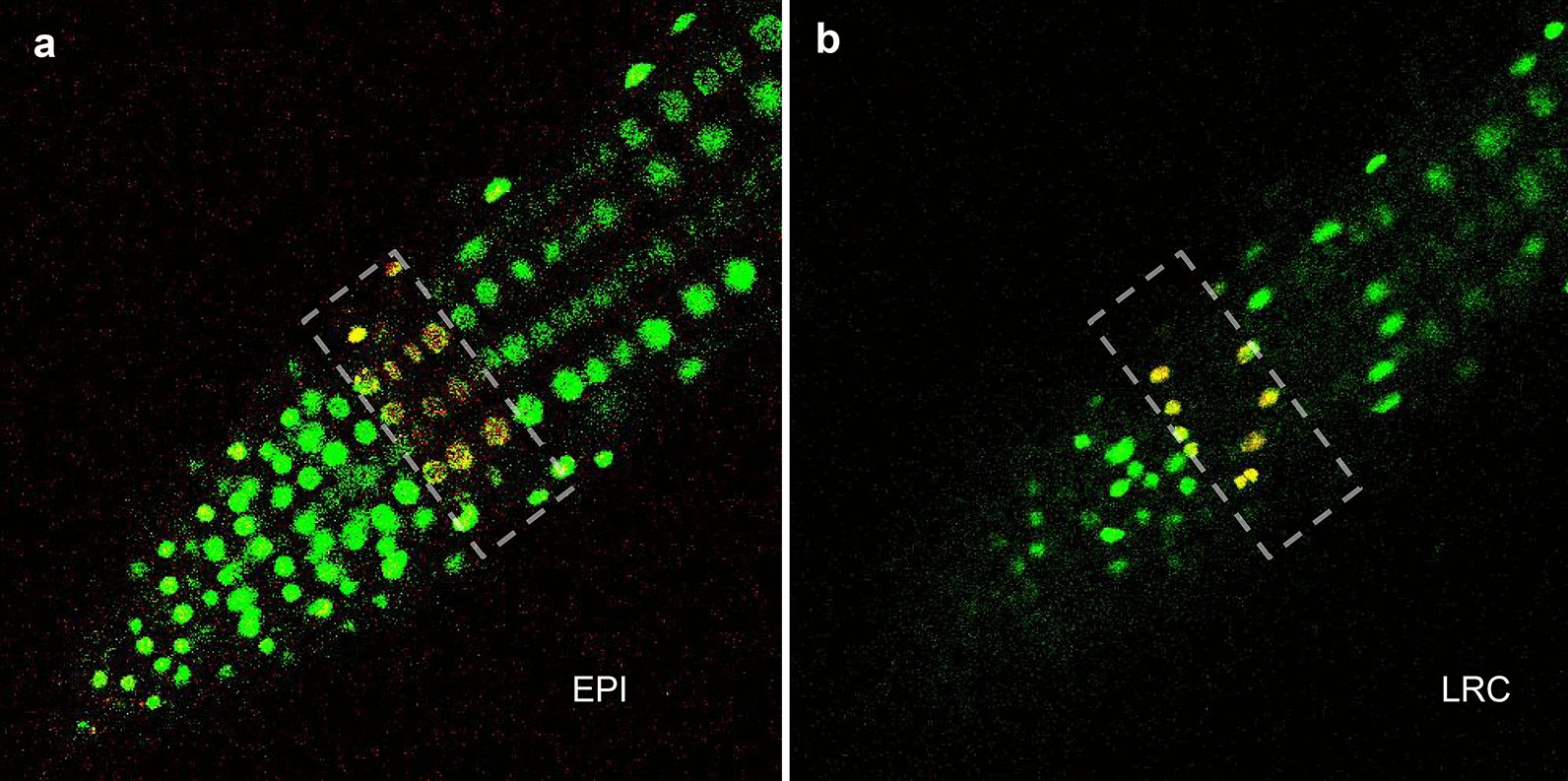
Photo-conversion of H2B-Dendra2 in the epidermis (EPI) and lateral root cap (LRC) of the primary root meristem. The dotted lines indicate the region of interest for photoconversion. The unconverted Dendra2 is shown in green and imaged (10% power of the 488 nm laser with 900 V master gain); and photoconverted Dendra2 is red and was imaged (30% power of the 561 nm laser with 1000 V master gain)

To test the functionality of Dendra2, we expressed H2B-Dendra2 in root epidermis using the WER promoter. To photoconvert Dendra, we used the “Bleaching” mode on a Ziess LSM 710 confocal; 60 iterations of 405 nm laser (8% of the full power) were performed prior to imaging. The green and red signals were then collected by the dual-channel (GFP/mCherry) [5]. The converted Dendra2 (now red) was clearly seen in the selected regions indicated by the enclosed dotted lines in Fig. 6. The selective photo-conversion of Dendra2 can be used not only to study protein dynamics but also to monitor plant growth over time. For example, H2B::mEosFP was previously exploited to assess changes in nuclear DNA content during the cell cycle in Arabidopsis [4].

## Discussion

### Compatibility analysis of gateway-compatible vectors

The root is a nearly ideal system for cell and developmental biology. It has clear radial patterning with stele in the innermost surrounded by one layer of endodermis and one layer of cortex. Outside of these cell layers lays the epidermis and lateral root cap. Several promoters have been identified to express specifically in certain cell types in Arabidopsis root. Promoter of *SHORT*-*ROOT* (*pSHR*) is expressed in stele cells and promoter *EN7* and *CO2* (*pEN7* and *pCO2*) have been found to be active in cortex and endodermis respectively [10, 13]. *WEREWOLF* promoter (*pWER*) has been shown to express specifically in epidermis [14]. *WOX5* gene (*pWOX5*) expression is in the quiescent center [15]. However, to express various genes under those promoters using traditional cloning mediated by digestion and ligation is laborious and sometimes difficult due to lack of digestion sites. Gateway cloning technology, which relies on recombination, allows swapping DNA fragment into the vector in a digestion-independent manner. Particularly multisite gateway cloning technology allows three entry clones, containing promoters, gene of interest and FPs respectively, recombine into a destination vector simultaneously. However, the efficiency of three-way recombination is low and high-efficient competent *E. coli* and expensive LR clonase II plus are indispensable. In many situations, even commercial competent cells do not guarantee successful recombination. Gel-purified entry vectors and freshly isolated destination vectors are often required, which makes the cloning process tedious. To facilitate high-throughput expression of genes in different cells, we established a gateway compatible cloning system to express fluorescent fusion proteins under cell-type specific promoters. Our destination vectors contain an attR1/attR2 gateway cassette with cell-type specific promoters (*pSHR, pEN7, pCO2, pWER* and *pWOX5*) at upstream the cassette and FPs (YFP, mCherry, CFP, Dendra2 and eGFP) at downstream the cassette (Fig. 3). These destination vectors can be used for a wide range of purposes including protein localization, protein function analysis, protein dynamics, protein movement and protein/protein interactions. As we include a multi-cloning site at the 5′ end of gateway cassette, the vectors can also be easily modified by adding other promoters that are active in leaves, flowers and other tissues. Here we provide 5 tissue-specific expression promoter vectors in roots. At the same time, 5 types of FPs-tagged vectors with multiple cloning sites at the upstream the cassette are provided, which can provide users with convenience in constructing different specific expression vectors in other tissues of plants.

## Conclusions

Here we reported the construction of Gateway (Invitrogen) compatible cloning system for cell-specific expression in roots of *Arabidopsis thaliana*. Our system alleviates the need for 3-way recombination, reduces the chances of transgene silencing and is adaptable for use in tissues outside of the *Arabidopsis* root. The relatively low cost of the system and ease of use facilitates the comparative study of protein localization, protein functions, protein dynamics and protein interactions in a cell-specific manner. Although presented the results using H2B and YFP in this manuscript, pSWU vectors have also been used in a variety of other functional proteins [16–20]. We believe our system can be applied to all different cloning strategies and simply modified for various expression purposes.

## Methods

### Vector construction

To generate the pSWU vectors, the full Gateway cassette—attR1/attR2 sites flanking the ccdB and CmR genes was amplified from pMDC7 in two steps. In the first step, two primers with partial MCS adapters were used to amplify the Gateway cassette. The PCR product was then reamplified using a second pair of primers containing the rest of MCS. The primers are listed in Additional file 3: Table S1. The resulting PCR product was then inserted into KpnI and HindIII digested pGreenBarT vector to replace the attR4/attR3 gateway cassette in pGreenBar. To introduce the various FPs into the pSWU vectors, YFP, mCherry, CFP, Dendra2 and erGFP coding sequences were amplified FROM using primer pairs that are listed in Additional file 3: Table S1. The amplified FP sequences were digested with XbaI and HindIII and then ligated into the pSWU vectors. The resulting vectors were named pSWU011 (YFP), pSWU012 (mCherry), pSWU013 (CFP), pSWU014 (Dendra2), pSWU015 (erGFP) (Fig. 3). Tissue specific promoters: *pSHR*, *pEN7*, *pCO2*, *pWER* were amplified and ligated into MCS-1of each of the SWU vectors using the KpnI/XhoI restriction sites. The *pWOX5* was ligated into the MCS-1 via KpnI/XhoI used In-Fusion cloning technique. The primers used to amplify the promoter sequences are listed in Additional file 3: Table S1. The resulting vectors were then renumbered as indicated in figure. All plasmid maps were included in Additional file 4.

### Testing the SWU vectors with H2B

H2B was amplified from *Arabidopsis thaliana* genomic DNA and recombined into pDONR221 using standard Gateway protocols (Invitrogen). The resulting entry clone was then recombined into the various destination vectors. In brief, 2–5 μl entry clone plasmid DNA and 1–2 μl destination vector plasmid DNA mix with 1–2 μl LR clonase II (5X). The reaction mixture was brought to a final volume of 5 μl or 10 μl with TE buffer (pH 8.0) and incubated at room temperature for 1–6 h before the transformation. After the LR reaction, and Proteinase K treatment the plasmid was transformed into DH5α for selection and amplification.

### Plasmid DNA amplification and isolation

The *E. coli* strain, DB3.1 (reference) was used for cloning and amplification of the pSWU vectors containing the intact gateway cassette (ccd-CM). Transformed bacteria were selected on solid LB media containing 30 μg/ml chloramphenicol and 100 μg/ml spectinomycin. For plasmid isolation, 2 ml of overnight culture was spun down and the pellet was resuspened in a 250 ml solution containing mM EDTA, 25 mM Tris, pH 8.0. After a brief incubation 250 ml solution of 0.2 N NaOH with 1% SDS was added followed by 350 ml of 3 M KOAc, pH 6.0. After centrifuge for 10 min, the supernatant was transferred to a new tube and mixed with and equal volume (700 µl) of isopropanol. The mixture was centrifuged for 30 min to pellet the plasmid DNA. 500 µl of 70% ethanol was used to wash the DNA pellet. After air drying for 20 min, the pellet was dissolved in ddH_2_O.

### Agrobacterium mediated transformation

All of the H2B destination vectors were transformed into Agrobacterium strain, GV3101-pSoup-pMP. 5 µl of plasmid was mixed with 50 µl chemically competent Agrobacterium (prepared by what method-citation) and incubated on ice for 30 min. The mixture was then heat-shocked at 37 °C for 5 min followed by incubation on ice for 10 min. After incubation on ice, 500 µl of LB medium was added and the bacterial cells were incubated for 2 h at 30 °C on a shaking incubator. The bacterial cultures were spread onto solid LB medium with antibiotics (50 μg/ml rifampicillin, 100 μg/ml spectinomycin, 30 μg/ml gentamycin and 5 μg/ml tetracycline) for selection. The transformed cells were grown at 28 °C for 2–3 days until colonies appeared. The Agrobacterium strains were used to transform Arabidopsis (Col-0) following the floral dip method [21]. Transgenic plants were screened by using resistance to glufosinate-ammonium (Basta) in soil.

### Plant materials and growth condition

*Arabidopsis thaliana* Columbia line (Col-0) was used as the wild type throughout the experiments. Plants were germinated and grown vertically on 0.5× MS medium (Caisson, www.caissonlabs.com) containing 0.05% (w/v) Mes (pH5.7), 1.0% (w/v) Sucrose, and 1% Granulated agar (DIFCO, www.bd.com) in a growth chamber at 23 °C under 16 h light/8 h dark cycle. Plants were imaged 5–6 days after plating unless otherwise stated.

### Confocal microscopy

Arabidopsis seedlings with intact roots were counterstained in 0.01 μg/ml propidium iodide (PI) and then placed on slides in a drop of water for confocal imaging. Confocal images were collected on a Leica TCS SL microscope using a 20× water-immersion lens or a Zeiss LSM 710 laser scanning confocal microscope using a Zeiss LD C-Apochromat 40×/1.1 NA water immersion objective lens (Carl Zeiss Microimaging Inc.). To convert Dendra2, we followed the previously described method [11]. In brief, on the Ziess LSM 710 confocal, we used the bleaching mode with ZEN 2009 software (Carl Zeiss Microimaging Inc.) with 10% of the 405 nm laser power. The iteration for photo-conversion was 60X in both epidermis and LRC. The pixel dwelling time for photoconversion was set to 1 µs. The green and red fluorescence of Denda2 was observed using 10% power of the 488 nm laser with 900 V master gain and 30% power of the 561 nm laser with 1000 V master gain.

## Supplementary information

**Additional file 1: Figure S1**. Previously reported *35S:SIEL*-*YFP* often shows transgene silencing in the meristem. Dotted line and arrow heads indicate the silenced cells.

**Additional file 2: Figure S2.** The Gateway-compatible vectors of* pWOL.* (a) The vectors shown in A were further modified by adding *pWOL* into MCS-1 at 5′ terminus of the gateway cassette. (b) The confocal iamges are *pWOL:erGFP*.

**Additional file 3: Table** **S1**. List of primers used for cloning.

**Additional file 4:** Maps of all constructed plasmids in this paper.

## Data Availability

The datasets used and/or analysed during the current study are available from the corresponding author on reasonable request.

## Abbreviations

FPs: Fluorescent proteins
CFP: Cyan fluorescing proteins
GFP: Green fluorescing proteins
YFP: Yellow fluorescing proteins
RFP: Red fluorescing proteins
BIFC: Bimolecular fluorescent complimentary
MCS: Multiple cloning sites

## References

[1] Shaner NC, Steinbach PA, Tsien R (2005). A guide to choosing fluorescent proteins. Nat Methods.

[2] Gurskaya NG, Verkhusha VV, Shcheglov AS, Staroverov DB, Chepurnykh TV, Fradkov AF, Lukyanov S, Lukyanov KA (2006). Engineering of a monomeric green-to-red photoactivatable fluorescent protein induced by blue light. Nat Biotech..

[3] Martin K, Kopperud K, Chakrabarty R, Banerjee R, Brooks R, Goodin MM (2009). Transient expression in *Nicotiana benthamiana* fluorescent marker lines provides enhanced definition of protein localization, movement and interactions in planta. Plant J..

[4] Mathur J, Radhamony R, Sinclair AM, Donoso A, Dunn N, Roach E, Radford D, Mohaghegh PM, Logan DC, Kokolic K, Mathur N (2010). mEosFP based green to red photoconvertible subcellular probes for plants. Plant Physiol.

[5] Wu S, Koizumi K, Macrae-Crerar A, Gallagher KL (2011). Assessing the utility of photo switchable fluorescent proteins for tracking intercellular protein movement in the *Arabidopsis* root. PLoS ONE.

[6] Jásik J, Boggetti B, Baluška F, Volkmann D, Gensch T, Rutten T, Altmann T, Schmelzer E (2013). PIN2 Turnover in *Arabidopsis* root epidermal cells explored by the photoconvertible protein Dendra2. PLoS ONE.

[7] Walter M, Chaban C, Schütze K, Batistic O, Weckermann K, Näke C, Blazevic D, Grefen C, Schumacher K, Oecking C, Harter K, Kudla J (2004). Visualization of protein interactions in living plant cells using bimolecular fluorescence complementation. Plant J..

[8] Citovsky V, Gafni Y, Tzfira T (2008). Localizing protein-protein interactions by bimolecular fluorescence complementation in planta. Methods.

[9] Schütze K, Harter K, Chaban C (2009). Bimolecular fluorescence complementation (BiFC) to study protein-protein interactions in living plant cells. Methods Mol Biol.

[10] Heidstra R, Welch D, Scheres B (2004). Mosaic analyses using marked activation and deletion clones dissect Arabidopsis SCARECROW action in asymmetric cell division. Genes Dev.

[11] Curtis Mark, Grossniklaus Ueli (2003). A Gateway TM cloning vector set for high-throughput functional analysis of genes in plants. Plant Physiol.

[12] Lee JY, Colinas J, Wang JY, Mace D, Ohler U, Benfey PN (2006). Transcriptional and posttranscriptional regulation of transcription factor expression in *Arabidopsis* roots. Proc Natl Acad Sci USA..

[13] Helariutta Y, Fukaki H, Wysocka-Diller J, Nakajima K, Jung J, Sena G, Hauser MT, Benfey PN (2000). The SHORT-ROOT gene controls radial patterning of the *Arabidopsis* root through radial signaling. Cell.

[14] Lee MM, Schiefelbein J (1999). WEREWOLF, a MYB-related protein in Arabidopsis, is a position-dependent regulator of epidermal cell patterning. Cell.

[15] Sarkar AK, Luijten M, Miyashima S, Lenhard M, Hashimoto T, Nakajima K, Scheres B, Heidstra R, Laux T (2007). Conserved factors regulate signalling in *Arabidopsis thaliana* shoot and root stem cell organizers. Nature.

[16] Wu S, Lee CM, Hayashi T, Price S, Divol S, Henry S, Pauluzzi G, Perin C, Gallagher KL (2014). A plausible mechanism, based upon SHORT-ROOT movement, for regulating the number of cortex cell layers in roots. Proc Natl Acad Sci U S A..

[17] Wu S, O’Lexy R, Xu M, Sang Y, Chen X, Yu Q, Gallagher KL (2016). Symplastic signaling instructs cell division, cell expansion, and cell polarity in the ground tissue of *Arabidopsis thaliana* roots. Proc Natl Acad Sci U S A..

[18] Yu Q, Li P, Liang N, Wang H, Xu M, Wu S (2017). Cell-fate specification in arabidopsis roots requires coordinative action of lineage instruction and positional reprogramming. Plant Physiol.

[19] Liu Y, Xu M, Liang N, Zheng Y, Yu Q, Wu S (2017). Symplastic communication spatially directs local auxin biosynthesis to maintain root stem cell niche in Arabidopsis. Proc Natl Acad Sci USA..

[20] Pengxue L, Qiaozhi Y, Xu G (2018). Construction of a functional casparian strip in non-endodermal lineages is orchestrated by two parallel signaling systems in, *Arabidopsis thaliana*. Curr Biol.

[21] Clough SJ, Bent AF (1998). Floral dip: a simplified method for Agrobacterium-mediated transformation of *Arabidopsis thaliana*. Plant J..

