# Supplementary figures and images for: Gateway-compatible vectors for functional analysis of proteins in cell type specific manner
Source: Plant Methods. 2020 Jul 6;16:93. doi: 10.1186/s13007-020-00635-z (PMC7339564; doi:10.1186/s13007-020-00635-z)

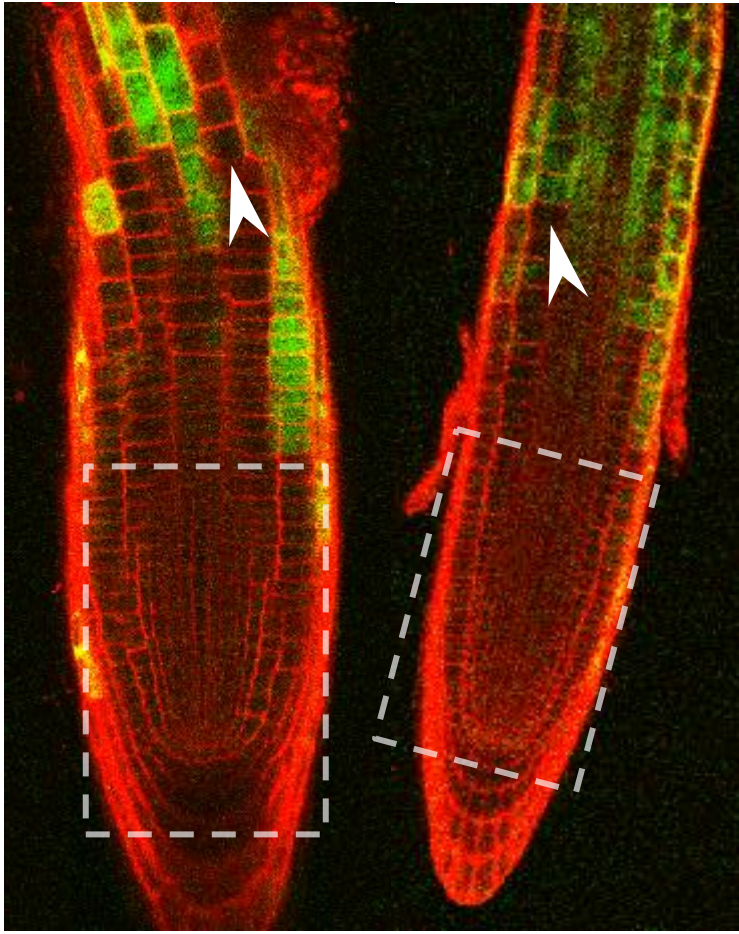

Supplement: Supplementary file 1 — Additional file 1: Figure S1. Previously reported 35S:SIEL-YFP often shows transgene silencing in the meristem. Dotted line and arrow heads indicate the silenced cells. [file 13007_2020_635_MOESM1_ESM.pdf]

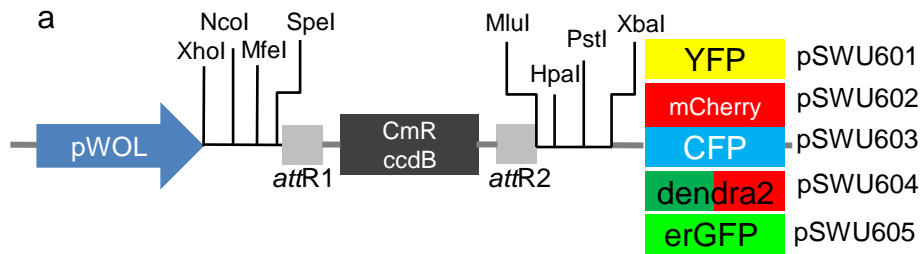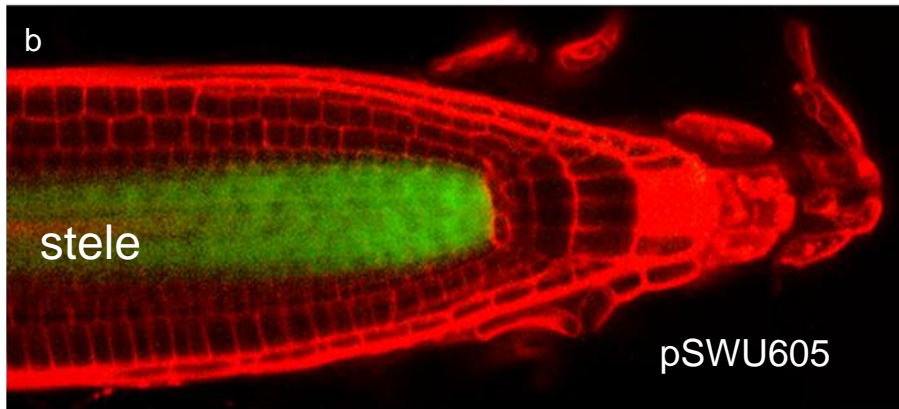

Supplement: Supplementary file 2 — Additional file 2: Figure S2. The Gateway-compatible vectors of pWOL. (a) The vectors shown in A were further modified by adding pWOL into MCS-1 at 5′ terminus of the gateway cassette. (b) The confocal iamges are pWOL:erGFP. [file 13007_2020_635_MOESM2_ESM.pdf]
