# Supplementary material for: Gateway-compatible vectors for functional analysis of proteins in cell type specific manner
Source: Plant Methods. 2020 Jul 6;16:93. doi: 10.1186/s13007-020-00635-z (PMC7339564; doi:10.1186/s13007-020-00635-z)
Supplement: Supplementary file 3 — Additional file 3: Table S1. List of primers used for cloning. [file 13007_2020_635_MOESM3_ESM.doc]

Supple Table 1. List of primers used for cloning

| name | 5’ to 3’ of sequence |
| --- | --- |
| Xbal-CFP-F | GCTCTAGAATGAGCAAGGGCGCCGAG |
| CFP-HindIII-R | GGAAGCTTACTTGTACAGCTCATCCATGC |
| Xbal-dendra2-F | GCTCTAGAATGAACACTCCTGGAATCAATC |
| dendra2-HindIII-R | GGAAGCTTAACCTGAGTCTCCAGACCAAAC |
| Xbal-mCherry/YFP-F | GCTCTAGAATGGTGAGCAAGGGCGAG |
| mCherry-HindIII-R | GGAAGCTTACTTGTACAGCTCATCCATGC |
| YFP-HindIII-R | GGAAGCTTAGATAGATCTCTTGTACAGCAGCTCG |
| Xbal-eGFP-F | TGCAGACGTTCTAGAATGGTGAGCAAGGGCGAGGA |
| eGFP-HindIII-R | CGTACCGATAAGCTTTTACTTGTACAGCTCGTCCATGCC |
| pEN7-Kpnl-F | ggcgaattgGGTACCCATGATCAGAGTATTGGGCCT |
| pEN7-Xhol-R | TTGCCATGGCTCGAGTTTAAGATTCTGAGATTCACGAAG |
| pCO2-Kpnl-F | ggcgaattgGGTACCCATGATCAGAGTATTGGGCCT |
| pCO2-Xhol-R | TTGCCATGGCTCGAGTATCGTTATTAACTAGGGTTCTTG |
| pSHR-KpnI-F | ggcgaattgGGTACCTTTCCTAGAATGCAAGAACAGA |
| pSHR-Xhol-R | TTGCCATGGCTCGAGTTTTTTTTTTTAATGAATAAGAAAATG |
| pWER-KpnI-F | ggcgaattgGGTACCAGGGGAGAGATGACTTCTTCTG |
| pWER-Xhol-R | TTGCCATGGCTCGAGTCTTTTTGTTTCTTTGAATGATAGA |
| pWOX5-KpnI-F | ggcgaattgGGTACCGAAAGACTTTTATCTACCAACTTCA |
| pWOX5-Xhol-R | TTGCCATGGCTCGAGGTTCAGATGTAAAGTCCTCAACTG |
| pWOL-Kpnl-F | ggcgaattgGGTACCTTTAATGATATAAAGTCAAAC |
| pWOL-Xhol-R | TTGCCATGGCTCGACACTTCAAATGTAGGTATTC |
| MCS1-2-KpnI-F | TCAGGTACCGGGCCCCCCCTCGAGCCATGGCAATTG |
| MCS1-attR1-F | CCCTCGAGCCATGGCAATTGACTAGTTATCAACAAGTTTGTACAAAAAAG |
| MCS2-attR2-R | GGATCCGGATATCTAGAACGTCTGCAGTTAACGCGTACCACTTTGTACAAGAAAGC |
| MCS2-2-HindIII-R | CGATAAGCTTCCCGGGATCCGATATCTAGAACG |
